# Nanosuspension delivery of paclitaxel to xenograft mice can alter drug disposition and anti-tumor activity

**DOI:** 10.1186/1556-276X-9-156

**Published:** 2014-04-01

**Authors:** Po-Chang Chiang, Stephen Gould, Michelle Nannini, Ann Qin, Yuzhong Deng, Alfonso Arrazate, Kimberly R Kam, Yingqing Ran, Harvey Wong

**Affiliations:** 1 Department of Pharmaceutics, Genentech, Inc., 1 DNA Way, South San Francisco, CA 94080, USA; 2 Department of In-Vivo Pharmacology, Genentech, Inc., 1 DNA Way, South San Francisco, CA 94080, USA; 3 Department of Drug Metabolism and Pharmacokinetics, Genentech, Inc., 1 DNA Way, South San Francisco, CA 94080, USA

**Keywords:** Paclitaxel, Nanosuspension, Cremophor EL, Pharmacokinetics, Tissue distribution, Xenograft

## Abstract

Paclitaxel is a common chemotherapeutic agent that is effective against various cancers. The poor aqueous solubility of paclitaxel necessitates a large percentage of Cremophor EL:ethanol (USP) in its commercial formulation which leads to hypersensitivity reactions in patients. We evaluate the use of a crystalline nanosuspension versus the USP formulation to deliver paclitaxel to tumor-bearing xenograft mice. Anti-tumor efficacy was assessed following intravenous administration of three 20 mg/kg doses of paclitaxel. Paclitaxel pharmacokinetics and tissue distribution were evaluated, and differences were observed between the two formulations. Plasma clearance and tissue to plasma ratio of mice that were dosed with the nanosuspension are approximately 33- and 11-fold higher compared to those of mice that were given the USP formulation. Despite a higher tumor to plasma ratio for the nanosuspension treatment group, absolute paclitaxel tumor exposure was higher for the USP group. Accordingly, a higher anti-tumor effect was observed in the xenograft mice that were dosed with the USP formulation (90% versus 42% tumor growth inhibition). This reduction in activity of nanoparticle formulation appeared to result from a slower than anticipated dissolution *in vivo*. This study illustrates a need for careful consideration of both dose and systemic solubility prior utilizing nanosuspension as a mode of intravenous delivery.

## Background

Paclitaxel is a chemotherapeutic agent used for the treatment of cancers. It acts by interfering with a cell's microtubule function by stabilizing microtubule formation, thereby inhibiting mitosis and normal cell division. Paclitaxel shows broad anti-tumor activity and is used to treat a wide variety of cancers such as ovarian, breast, non-small cell lung, head and neck cancer, and advanced forms of Kaposi's sarcoma [1-4]. Despite its broad use as a chemotherapeutic, the delivery of paclitaxel is challenging. Paclitaxel is a well-known BCS class IV drug with poor solubility and poor permeability which serves to limit its oral uptake. Also, paclitaxel is a substrate of the membrane-bound drug efflux pump P-glycoprotein (P-gp), which can prevent oral absorption or uptake by mediating direct excretion of the drug into the intestinal lumen [1,5]. Finally, significant pre-systemic first-pass metabolism in the liver by the cytochrome P450 enzymes further reduces the oral bioavailability of paclitaxel [6-8].

As a result of the described challenges to oral delivery, the current route of paclitaxel administration is via the intravenous (IV) route. Due to its poor solubility, paclitaxel is dissolved in organic mix of Cremophor EL (BASF, Ludwigshafen, Germany):ethanol (1:1 *v*/*v*) for intravenous delivery. Cremophor EL is a polyoxyethylated castor oil, and its inclusion in the delivery vehicle has resulted in severe hypersensitivity reactions occurring after IV administration [9-15]. Anti-allergic pre-medication treatment with corticosteroids and antihistamines has been used to reduce the incidence of adverse reactions associated with paclitaxel. Despite pre-medication, milder hypersensitivity reactions still occur in 5% to 30% of patients [4]. The described liability highlights the need for a new formulation vehicle. Tween 80- and Tween 80/ethanol-based formulations with subsequent dilution using aqueous media have been tested for paclitaxel. In both cases, dilution with aqueous media resulted in precipitation of paclitaxel which was a major concern [16-19]. Liposome-based formulations have also been tested and have shown promise [20-22]. However, drawbacks for liposome formulations include rapid degradation due to the reticuloendothelial system (RES), an inability to achieve sustained drug delivery over a prolonged period of time [23], and low drug load which often limits their application. Thus, there is still a need to explore alternate formulations for paclitaxel and poorly soluble compounds in general.

Recently, the use of nano- and microparticle drug delivery in the pharmaceutical industry has been reported. This formulation technology has been applied to a variety of dosing routes including the oral, intraperitoneal (IP), intramuscular (IM), inhalation, intratracheal (IT), intranasal (IN), and subcutaneous (SC) dosing routes, or to enable direct target delivery [24-28]. The main advantage of using nano- or microparticle delivery systems is that the small particle size creates an increased surface area which acts to enhance the overall dissolution rate, thereby improving the bioavailability of extravascular dosing routes without the use of solvents. The described advantage of an improved dissolution rate can also be applied to the IV route [28-34]. The use of nanoparticles for IV formulations has recently drawn much attention [28-34]. However, there is a need for more *in vivo* investigations evaluating intravenous delivery with nanoparticle formulations. The impact of intravenous nanosuspension delivery on pharmacokinetics, tissue/organ distribution, and pharmacodynamics/efficacy are not fully understood. The objective of our current study is to investigate the effect of intravenous nanosuspension delivery of paclitaxel to a xenograft mouse tumor model compared to the standard Cremophor EL:ethanol formulation. In particular, comparisons of pharmacokinetics, organ distribution, and anti-tumor effect were evaluated for both formulations following intravenous administration. We observe differences in paclitaxel pharmacokinetics, tissue distribution, and most importantly anti-tumor effect due to nanosuspension delivery.

## Methods

High-performance liquid chromatography (HPLC)-grade acetonitrile was obtained from Burdick & Jackson (Muskegon, MI, USA), reagent-grade formic acid was obtained from EM Science (Gibbstown, NJ, USA), and paclitaxel bulk drug was purchased from Sigma-Aldrich (St. Louis, MO, USA). Commercially available paclitaxel (Cremophor EL:ethanol) was manufactured by Bristol-Myers Squibb (New York, NY, USA). Other chemicals were either made in-house (Genentech, Inc., South San Francisco, CA, USA) or purchased from Sigma-Aldrich. The water purification system used was a Millipore Milli-Q system (Billerica, MA, USA).

### Powder X-ray diffraction pattern and particle size determination

Powder X-ray diffraction (PXRD) patterns were recorded at room temperature with a Rigaku (The Woodlands, TX, USA) MiniFlex II desktop X-ray powder diffractometer. Radiation of Cu Kα at 30 kV and −15 mA was used with 2*θ* increment rate of 3°/min. The scans were run over a range of 2° to 40° 2*θ* with a step size of 0.02° and a step time of 2 s. Powder samples were placed on a flat silicon zero background sample holder.

The particle size distribution of the nanosuspension was measured by using a Nanotrac (Montgomeryville, PA, USA) instrument. Triplicates were measured for each sample, and the average was used for the final particle size distribution. The particle size distribution was calculated based on the general purpose (normal sensitivity) analysis model and the following refractive indices (RIs): particle RI, 1.58; absorption, 1.0; and dispersant RI, 1.38.

### Formulation preparation for paclitaxel IV crystalline nanosuspension and stability evaluation

A bench scale wet milling method was developed for particle size reduction and has been previously described [33]. Briefly, a paclitaxel stock nanosuspension formulation (20 mg/mL) was prepared by mixing paclitaxel with an appropriate amount of glass beads and vehicle containing 0.1% (*w*/*w*) Cremophor EL in phosphate saline (pH 7.4) in a scintillation vial. The mixture was stirred at 1,200 rpm for a period of 24 h with occasional shaking. The resulting stock formulation was diluted to the target concentration with vehicle and then harvested. Paclitaxel concentrations were verified by a HPLC assay. Analysis of milled paclitaxel particles was performed using a Nanotrac (Montgomeryville, PA, USA) instrument. An assessment of form change in pre- and post-milling samples was performed using PXRD.

The rate of dissolution of paclitaxel in nanosuspension is expected to be higher compared to regular suspension due to the reduction of particle size. The Noyes and Whitney equation (Equation 1) was used in order to assess the impact of particle size reduction on dissolution rate and is described as follows:

(1) $$\mathrm{dC}/\mathrm{dt}\;=\;D\times S\;\left(C_{s}\;-\;C_{t}\left(t\right)\right)/Vh_{d}$$

where *dC*/*dt* is the dissolution rate, *D* is the solute diffusion coefficient, *V* is the volume of the dissolution medium, *h*_*d*_ is the diffusion boundary thickness, *S* is the surface area of the solute, *C*_*s*_ is the saturation solubility of the solute, and *C*_*t*_(*t*) is the bulk solute concentration. The stability of the crystalline nanosuspension was monitored for a period of 3 weeks for particle size, PXRD, and chemical stability (by HPLC). Paclitaxel plasma solubility was determined by adding excess amount of paclitaxel bulk drug into 0.5 mL of rodent plasma (obtained in-house) which was allowed to equilibrate at 37°C on a rotary shaker for a period of 24 h. Excess drug was then removed by centrifugation which was followed by protein precipitation, and the concentration was measured by HPLC with an external standard.

### Efficacy and pharmacokinetic study in xenograft mice

Briefly, 2.5 million Calu-3 non-small cell lung cancer cells were resuspended in Hank's balanced salt solution and implanted intradermally into the hind flank of female SCID-bg mice (Charles River Laboratories, Hollister, CA, USA). When tumor volumes reached approximately 150 to 300 mm^3^, mice were randomly assigned to three treatment groups. Treatment groups were administered one intravenous dose every 4 days of either vehicle (Cremophor EL:ethanol 1:1, saline; *n* = 10), paclitaxel formulated in Cremophor vehicle (*n* = 15), or paclitaxel formulated in nanosuspension (*n* = 15). A total of three doses were given during the course of the study. The paclitaxel dose was selected in an attempt to match as best possible clinically relevant exposures and at the same time provide robust anti-tumor efficacy when delivered with the commercial formulation (Cremophor EL:ethanol 1:1). Tumor volumes were measured in two dimensions (length and width) using Ultra Cal-IV calipers (Model 54-10-111, Fred V. Fowler Company, Inc., Newton, MA, USA). The following formula was used with Excel v11.2 (Microsoft Corporation, Redmond, WA, USA) to calculate tumor volume (TV): TV (mm^3^) = (length × width^2^) × 0.5. Tumor sizes and body weights were recorded twice weekly, and the mice were regularly observed over the course of the study. Mice were euthanized if their tumor volume exceeded 2,000 mm^3^ or if their body weight dropped by more than 20% of the starting weight. At end of the study, mice in both paclitaxel groups were given a final dose of paclitaxel, and blood (collected by terminal cardiac puncture and plasma-harvested) and tissues (liver, spleen, and tumor) were collected at various time points (10 min, 30 min, 2 h, 4 h, and 8 h post-dose). Three mice were taken down at each time point, and biological samples were frozen at −70°C until sampling. Paclitaxel concentrations in plasma and tissues were measured by a liquid chromatography tandem mass spectrometry (LC/MS/MS) assay. The study was conducted in accordance with the institutional guidelines for humane treatment of animals and was approved by the IACUC of Genentech.

### LC/MS/MS assay for the determination of paclitaxel

Concentrations of paclitaxel in mouse plasma, tumor, liver, and spleen were determined by a LC/MS/MS assay. Tumor, liver, and spleen tissue samples were diluted 4-fold with water and homogenized by using a FastPrep-24 bead beater (MP Biomedicals, Solon, OH, USA). The plasma and tissue homogenate samples were prepared for analysis by placing a 25-μL aliquot into a 96-well plate followed by the addition of 5 μL of internal standard (docetaxel, 2 μg/mL in 50:50, *v*/*v*, DMSO:water) and 200 μL acetonitrile. The samples were vortexed and centrifuged at 1,600 g for 15 min at room temperature, 50 μL of the supernatant was diluted with 150 μL of water, and 5 μL of the solution was injected onto a Kinetex XB C-18 (30 × 2.1 mm, 2.6 μm) analytical column (Phenomenex, Torrance, CA, USA).

An Agilent 1290 Infinity HPLC system (Agilent, Santa Clara, CA, USA) was equipped with a controller, two pumps, a column compartment, and a degasser. The column was maintained at 40°C by the column compartment. This system was coupled to an API 5500 Qtrap mass spectrometer (AB Sciex, Foster City, CA, USA) equipped with a turbo-electrospray interface in positive ionization mode. The aqueous mobile phase was water with 0.1% formic acid (A), and the organic mobile phase was acetonitrile with 0.1% formic acid (B). The gradient was as follows: starting at 15% B and increased to 95% B for 0.6 min, maintained at 95% B for 0.1 min, then decreased to 15% B within 0.1 min. The total flow rate was 1.4 mL/min. Data was collected using multiple reaction monitoring (MRM) with transitions *m*/*z* 854.4 → 104.9 for paclitaxel and *m*/*z* 808.5 → 527.2 for docetaxel (internal standard). The calibration curve, which ranged from 0.03 to 24 μM for paclitaxel, was fitted to a 1/*x* weighted quadratic regression model. This calibration curve was used to quantitate paclitaxel concentration levels in the plasma, tumor, liver, and spleen samples.

### Data analysis

Pharmacokinetic parameters were estimated by non-compartmental methods as described by Gibaldi and Perrier [35] using WinNonlin version 3.2 (Pharsight Corporation, Mountain View, CA, USA). Tissue to plasma ratios were determined by dividing the AUC_0-8_ (area under the concentration-time profile from 0 to 8 h) of the tissue of interest by the AUC_0-8_ of plasma.

The percent tumor growth inhibition (%TGI) was calculated on the last day of the study (day 17) using the following formula as previously described [36]:

(2) $$\%\mathrm{TGI}\;=\;\frac{{\mathrm{TV}}_{\mathrm{vehicle}}\;-\;{\mathrm{TV}}_{\mathrm{treatment}}}{{\mathrm{TV}}_{\mathrm{vehicle}}\;-\;{\mathrm{TV}}_{\mathrm{initial}}}\;\times \;100$$

TV_vehicle_ is the tumor volume for the vehicle-treated animals on day 17, TV_initial_ is the initial tumor volume at the start of the treatment, and TV_treatment_ is the tumor volume of the treatment groups on day 17. Normalized efficacy was determined with respect to plasma and tumor exposures for both Cremophor EL:ethanol and nanosuspension delivery. Normalized efficacy was determined by dividing TGI by either plasma or tumor AUC_0-8_.

## Results

### Formulation preparation for paclitaxel IV crystalline nanosuspension and stability evaluation

A theoretical calculation was performed to estimate the target particle size at which a nanoparticle should rapidly dissolve in the bloodstream (i.e., < 10 s under non-stirred condition) upon intravenous administration. In order to estimate the dissolution rate of the nanoparticles in blood upon IV administration, the Stokes-Einstein equation [33,34] was used to estimate the diffusion coefficient (for free diffusion assuming a spherical molecule):

$$D\;=\;\frac{\mathrm{kT}}{6\mathrm{\pi r\eta}}$$

where *k* is the Boltzmann constant (1.38 × 10^−23^ J/K), *η* is the solvent viscosity (kg/ms; for blood = 0.035 kg/ms), *T* is the temperature (K; 37°C), and *r* is the solute molecule radius (cm).

This equation can be extended to relate the diffusion coefficient to the molecular weight and density of the molecule of interest:

MW=NρV=Nρ43πr3andr=3MW4πNρ13

where *N* is Avogadro's number, *V* is the molar volume of the solute, *r* is the hydrodynamic radius, which considers the solvent bound to the solute, and *ρ* is the density of the solute.

The resulting equation is as follows:

D=kT6πη3MW4πNρ13

Using the MW for paclitaxel (MW = 853.9), the diffusion coefficient (*D*) was calculated to be 9.5 × 10^−7^ cm^2^/s. An estimate of the particle radius needed to achieve a dissolution time of <10 s under non-stirred sink condition was determined using the Hixson-Crowell cube root law [33,34]:

$$\Gamma =\rho \;{r_{o}}^{2}/2\mathrm{DCs}$$

where *Γ* is the estimate time for complete dissolution, *ρ* is the density of the solution, *r*_*o*_ is the radius of the particle, *D* is the diffusion coefficient, *Cs* is the solubility in plasma at 37°C (40 μg/mL).

**Figure 1:**
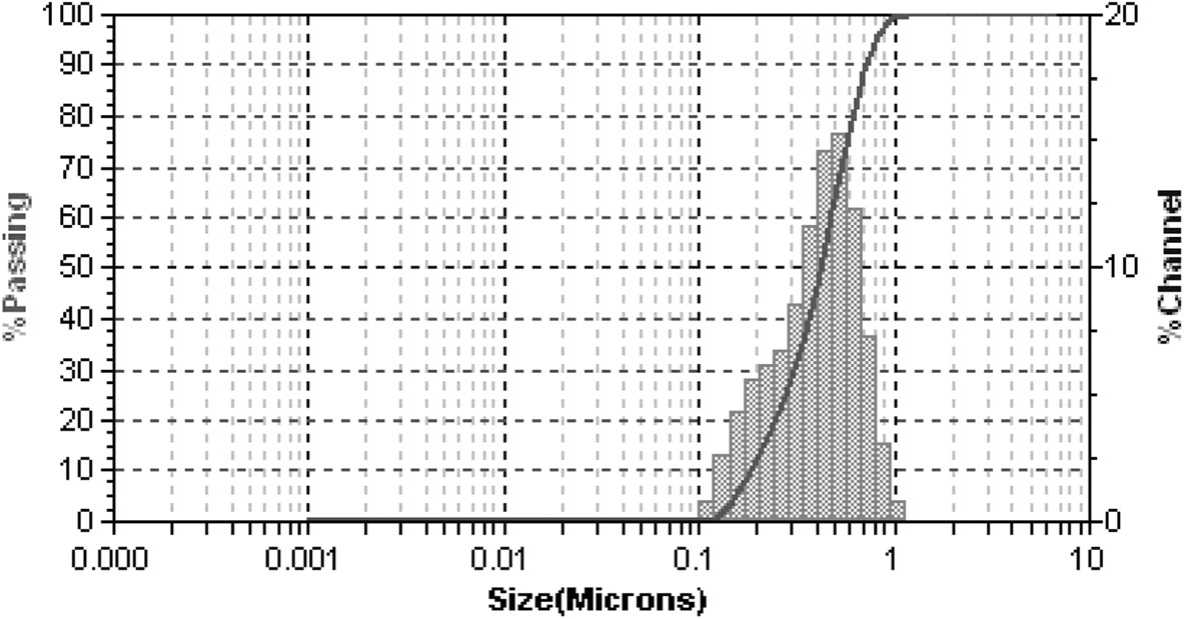
Particle size characterization of paclitaxel nanosuspension.

**Figure 2:**
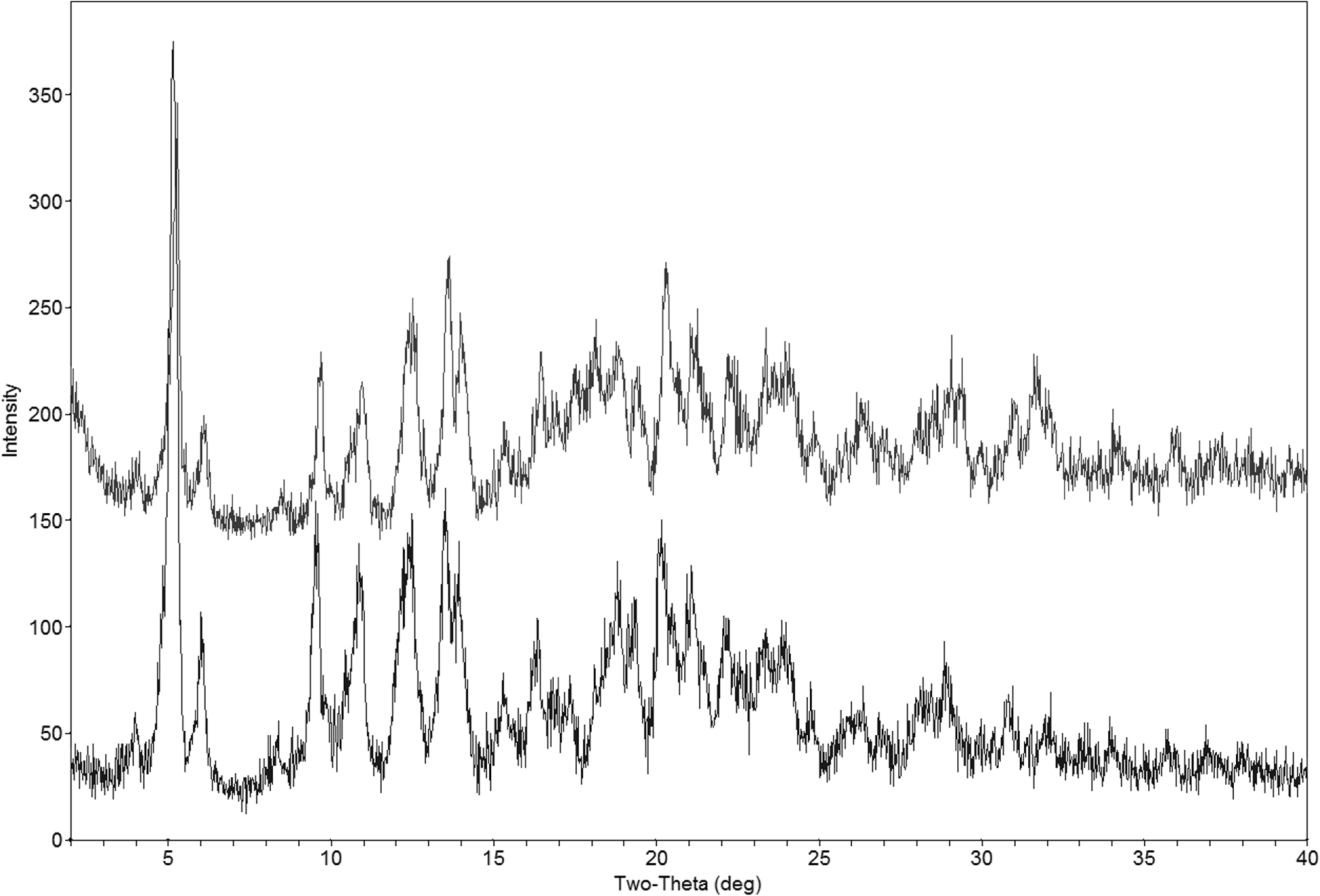
PXRD of paclitaxel post-milling (top) and API (bottom).

Based on the relationship described above, the calculated target mean radius for the paclitaxel nanoparticles was calculated to be 0.6 μm under sink conditions. The paclitaxel nanosuspension was characterized in order to ensure its proper preparation. *D*_50_ and *D*_90_ of paclitaxel particles in the IV formulation were determined to be 0.4 and 0.7 μm, respectively (Figure 1). A *D*_50_ of 0.4 μm was within the mean target radius of 0.6 μm. PXRD characterization of the solid form of the nanomaterial indicated no significant change in crystal form from the milling process (Figure 2). The paclitaxel crystalline nanosuspension formulation was stable at room temperature with no significant changes in PXRD, particle size, and chemical stability over a period of 3 weeks.

Using a previously published theoretical calculation [30,33,34], measured paclitaxel solubility in plasma (40 ± 2 μg/mL at 37°C), and the *D*_50_ listed above, the estimated dissolution time of an average paclitaxel particle in the nanosuspension was estimated to be less than 5 s. The actual *in vivo* dissolution time should theoretically be much more rapid since turbulent blood flow in the vein should serve to both reduce the diffusion boundary thickness and rapidly disperse the injection formulation minimizing local concentration effects [33,34].

### Plasma and tissue pharmacokinetics in tumor-bearing xenograft mice

**Figure 3:**
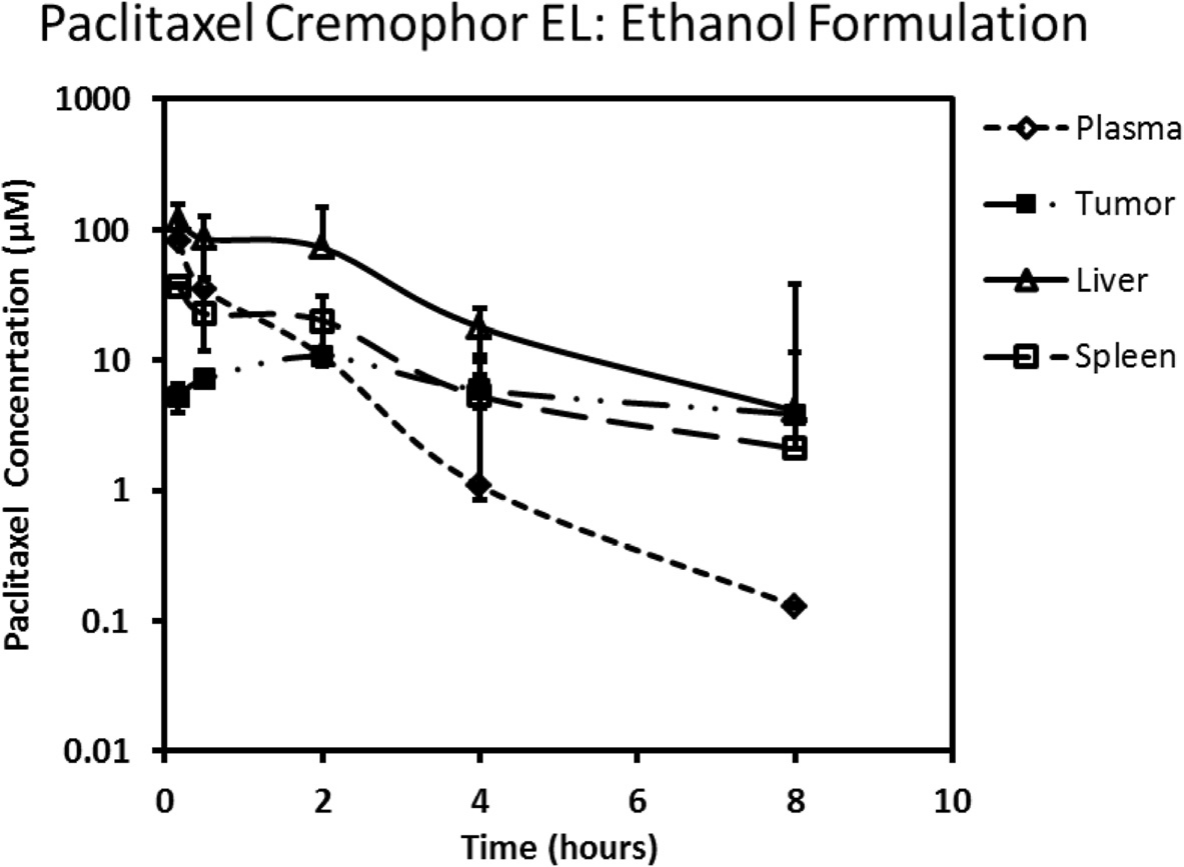
Paclitaxel concentration-time profile in plasma, tumor, liver, and spleen following intravenous administration using Cremophor EL:ethanol formulation.

**Figure 4:**
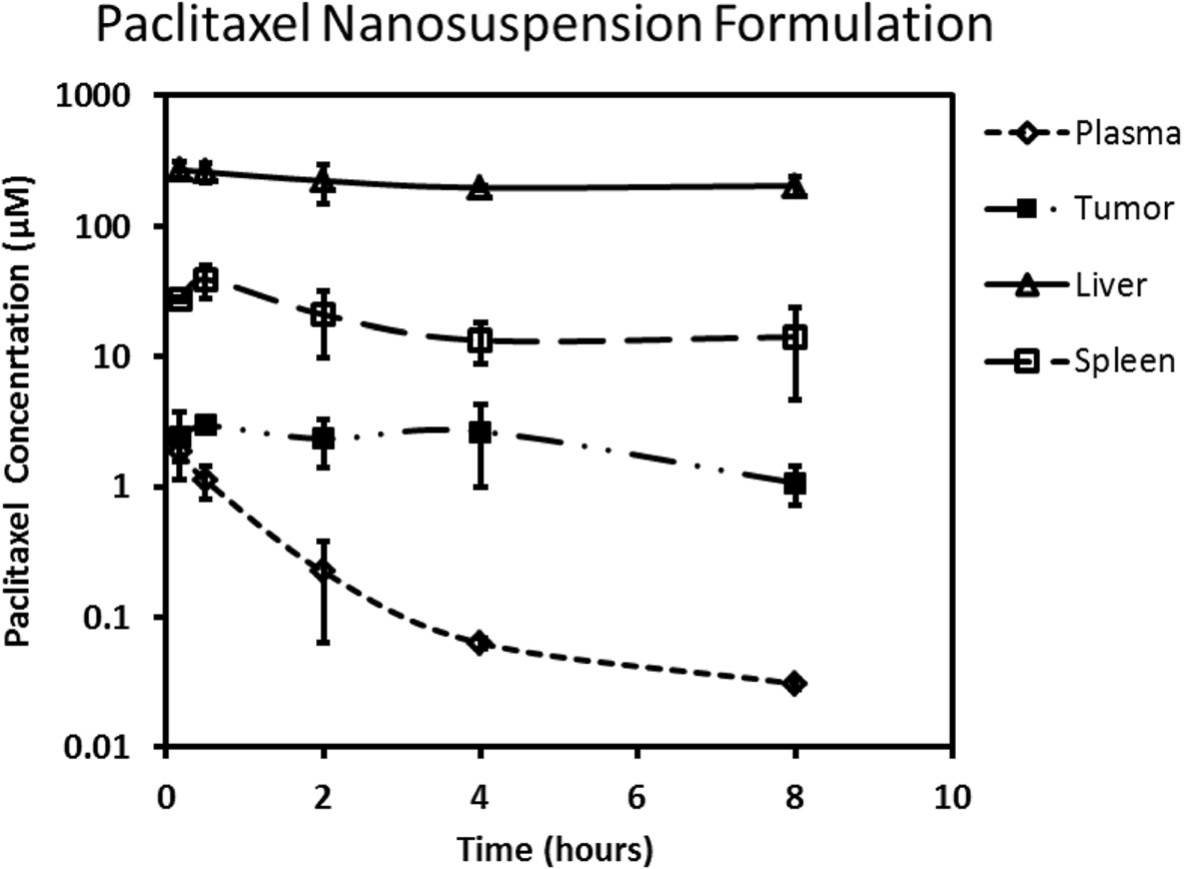
Paclitaxel concentration-time profile in plasma, tumor, liver, and spleen following intravenous administration using nanosuspension formulation.

Paclitaxel plasma, tumor, spleen, and liver concentration-time profiles following intravenous administration at 20 mg/kg using the Cremophor EL:ethanol and nanosuspension formulations are presented in Figures 3 and 4, respectively. The plasma clearance of paclitaxel after intravenous dosing was substantially higher with nanosuspension (158.3 mL/min/kg) delivery compared to the standard Cremophor EL:ethanol formulation (4.9 mL/min/kg). Accordingly, plasma AUC_0-8_ was approximately 40-fold less with nanosuspension delivery (Table 1). Tumor concentrations (Figures 3 and 4) and exposures (Table 1) were higher for Cremophor EL:ethanol delivery with AUC_0-8_ being approximately 3-fold higher compared to nanosuspension delivery. In contrast, paclitaxel liver concentrations (Figures 3 and 4) and exposures (Table 1) were higher for nanosuspension delivery with AUC_0-8_ being approximately 6-fold higher than that observed for the Cremophor EL:ethanol formulation. Spleen exposure was comparable for the two formulations (Table 1).

**Table 1:** Exposure (mean value) of paclitaxel in plasma, tumor, liver, and spleen following intravenous administration

**Tissue AUC**_**0**-**8**_**(μM × h)**	**Formulation**	
	**Cremophor EL:ethanol**	**Nanosuspension**
Plasma	74.7	2.1
Tumor	52.1	17.5
Liver	269.1	1,701.1
Spleen	85.2	147.5

**Figure 5:**
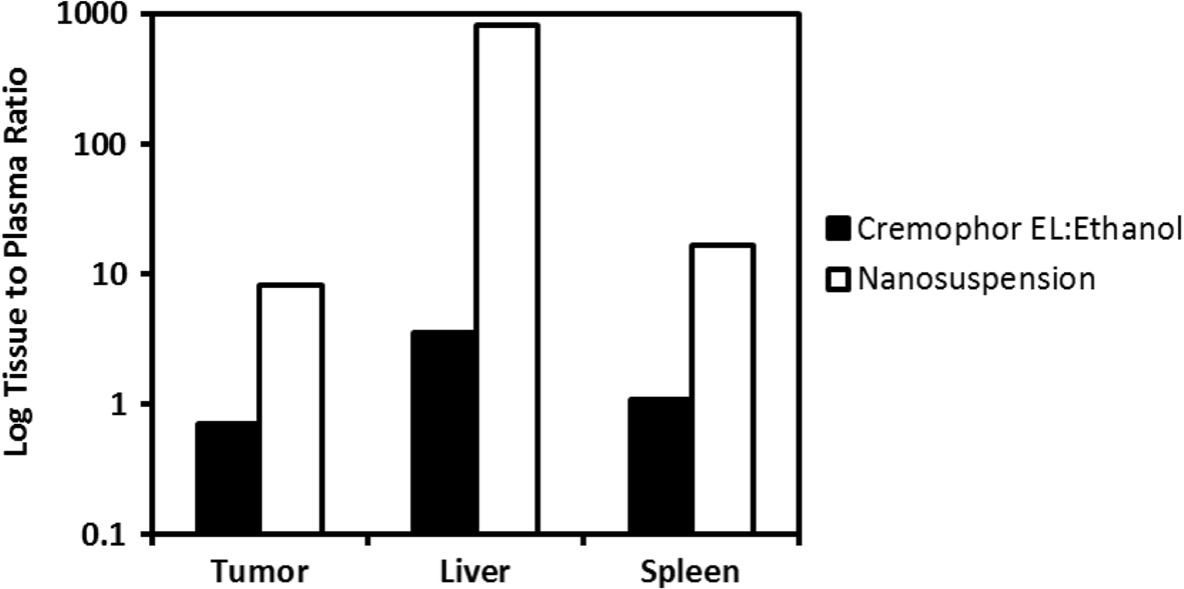
Log tissue to plasma ratios for tumor, liver, and spleen following intravenous delivery to mice.

Paclitaxel tissue to plasma ratios were determined in order to assess formulation-dependent differences in tissue distribution in tumor, spleen, and liver (Table 2). Delivery with nanosuspension resulted in higher tissue to plasma ratios for all three organs investigated (Figure 5, Table 2). In particular, the liver to plasma ratio was exceptionally high being approximately 225-fold higher with nanosuspension delivery.

**Table 2:** Tissue to plasma exposure ratio of paclitaxel for tumor, liver, and spleen following intravenous administration

**Tissue to plasma ratio**	**Formulation**	
	**Cremophor EL:ethanol**	**Nanosuspension**
Tumor AUC_0-8_/plasma AUC_0-8_	0.7	8.3
Liver AUC_0-8_/plasma AUC_0-8_	3.6	810.0
Spleen AUC_0-8_/plasma AUC_0-8_	1.1	16.8

AUC_0-8_, area under the concentration-time profile from 0 to 8 h.

### Anti-tumor efficacy of paclitaxel

**Figure 6:**
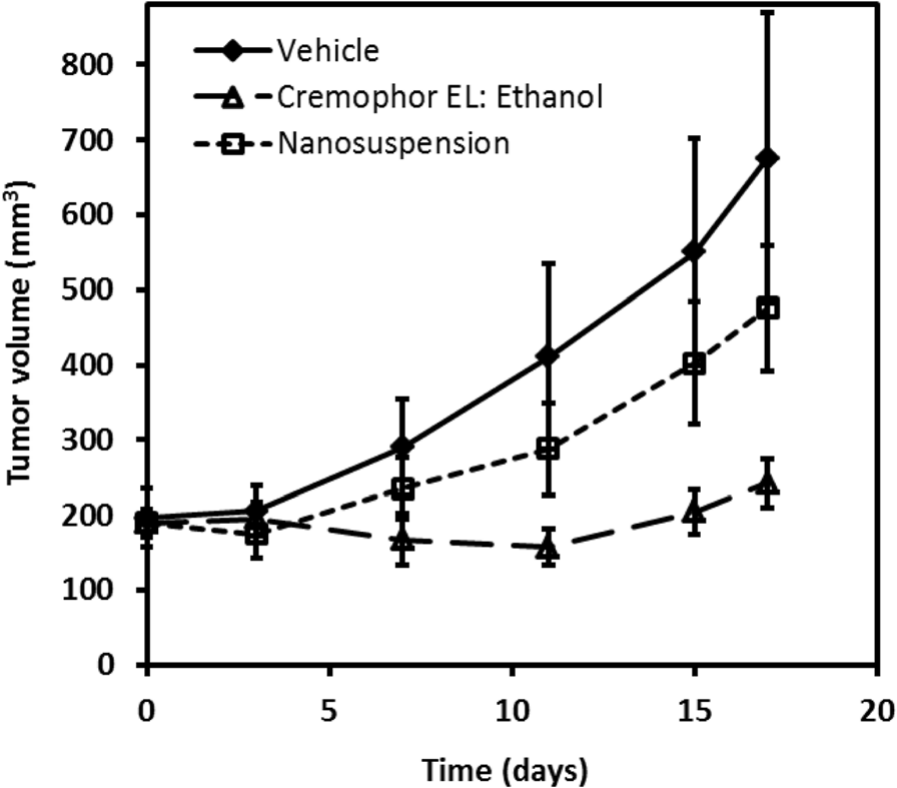
Plots of mean tumor volume versus time in xenograft mice for intravenous paclitaxel.

In order to compare the relative efficacy of Cremophor EL:ethanol versus nanosuspension delivery, percent tumor growth inhibition was determined at the end of the study. Delivery of paclitaxel with the standard Cremophor EL:ethanol formulation resulted in 90% TGI (Figure 6). The use of nanosuspension for intravenous delivery resulted in considerably less efficacy with TGI being 42%.

**Figure 7:**
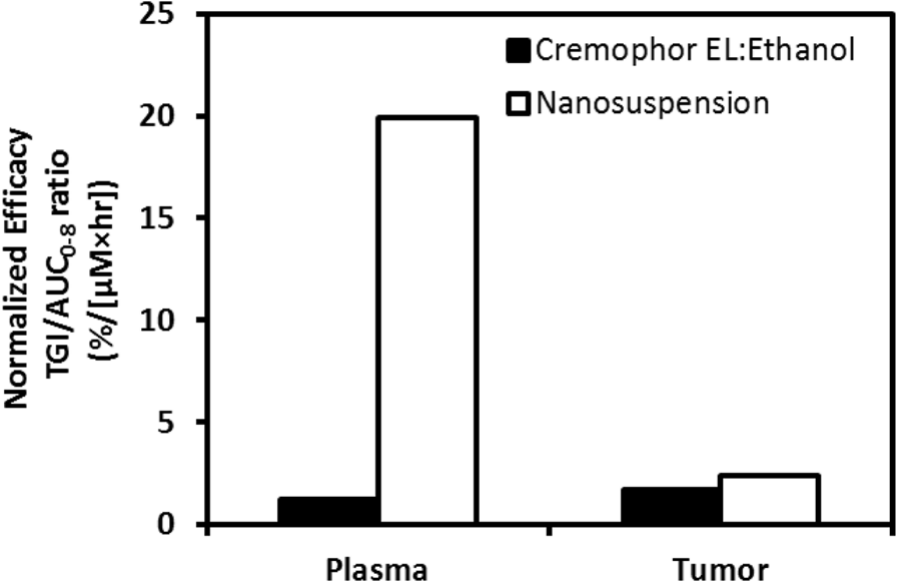
Normalized efficacy based on plasma and tumor concentrations following delivery of paclitaxel to xenograft mice.

In order to normalize the anti-tumor efficacy with differences in paclitaxel exposure observed with the two formulations, TGI was normalized with respect to the plasma and the site of action (i.e., tumor). Figure 7 shows normalized efficacy with respect to plasma and tumor exposures for both formulations. The normalized efficacy with respect to plasma was considerably higher for nanosuspension delivery being approximately 20-fold higher compared to delivery with Cremophor EL:ethanol. However, when efficacy was normalized with respect to tumor which is the site of action, there was little difference in normalized efficacy between the two formulations (Figure 7).

**Figure 8:**
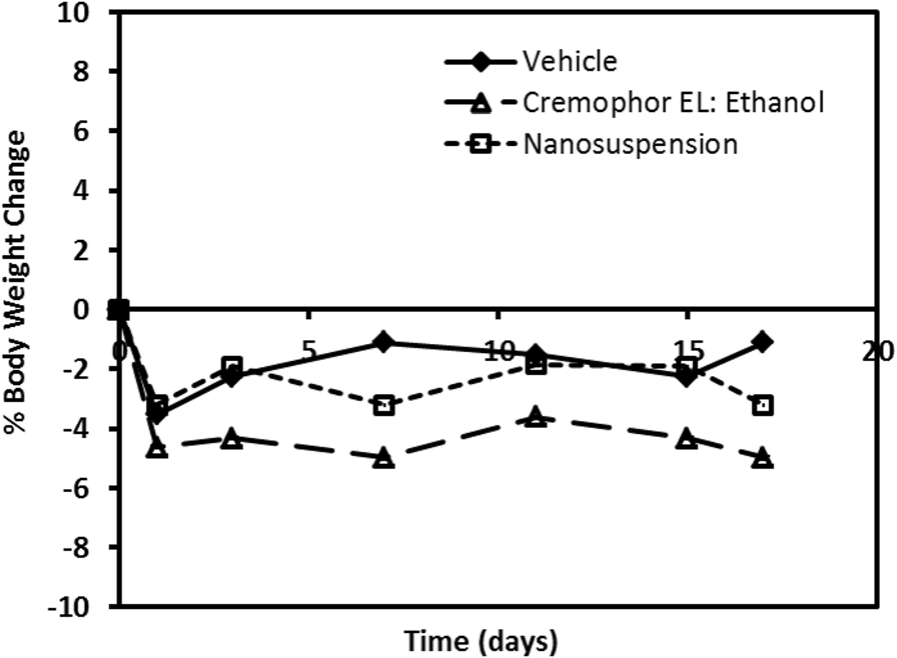
Mean percent body weight change in xenograft mice given intravenous paclitaxel.

Body weight changes were also monitored in the xenograft mouse efficacy study in order to give a crude assessment of formulation tolerability (Figure 8). There appeared to be no substantial differences in body weight changes when comparing the three treatment groups of mice.

## Discussion

Poorly soluble compounds are an increasing problem in the pharmaceutical industry. The oral and intravenous delivery of an increasing number of poorly soluble compounds for *in vivo* evaluation is a growing challenge for formulation scientists. For the oral delivery, particle size reduction of solid drug substance offers a means to increase the dissolution rate and improve oral bioavailability of poorly soluble compounds. As a result, the use of nanoparticles has been adapted as a formulation approach to improve the oral delivery of poorly soluble compounds [24,27]. Similarly, delivery by the intravenous route can also benefit from the use of nanoparticles since nanoparticle formulations offer the advantage of reducing the organic solvent content often required for poorly soluble compounds. The small particle size afforded by the use of nanoparticles should enable a rapid, almost instantaneous dissolution of solid particles following intravenous administration due to a high dissolution rate with blood acting as the dissolution media. However, there are particle size requirements for intravenous dosing since the completion of the dissolution process must be instantaneous due to potential risks such as phlebitis and undesired organ accumulation that may occur upon injection [34].

Paclitaxel is an extensively used chemotherapeutic agent that suffers from very poor solubility. As such, the commercial intravenous formulation of paclitaxel requires the inclusion of Cremophor EL in order to keep it solubilized. The use of Cremophor EL in the intravenous paclitaxel formulation has introduced a number of unique undesirable features including non-linear pharmacokinetics [37] and more importantly hypersensitivity reactions which require anti-allergic pre-medication with corticosteroids and antihistamines [4]. Due to these undesirable properties, there is a need to explore alternate formulations. We had previously evaluated the use of nanosuspension to enable intravenous delivery of ten poorly soluble compounds in a cassette dosing format [34]. In this intravenous cassette study, no changes in pharmacokinetics were observed with nanosuspension delivery compared to the use of a solution formulation with high organic content. In contrast, our current work with paclitaxel nanosuspension delivery shows substantial alterations in the pharmacokinetic properties of paclitaxel compared with the standard Cremophor EL formulation (Figures 3 and 4). Plasma clearance was substantially higher (approximately 30-fold) with nanosuspension delivery. Since paclitaxel was given intravenously, alterations in plasma pharmacokinetics are attributed entirely to alterations in paclitaxel distribution and/or systemic elimination. Distribution was clearly different with higher tissue to plasma ratios in the spleen, liver, and tumor following nanosuspension delivery (Figure 5, Table 2). In particular, a high concentration of paclitaxel was present in the liver. This high sustained concentration of paclitaxel in the liver may result in an overestimation of plasma clearance since plasma concentrations drop rapidly yet drug was not really eliminated from the body, but rather trapped in the liver. An explanation for the high concentrations of drug in tissue may be that the nanoparticles in the nanosuspension may be dissolving slower than anticipated *in vivo*. Our theoretical estimation of the required particle size for instantaneous dissolution was based on assumed sink conditions. We did not observe alterations in pharmacokinetics in our previous cassette doing study [34] with intravenous administration of ten poorly soluble compounds. However, in our previous study, low doses (0.5 mg/kg) of each compound were administered, and therefore, the assumption of sink conditions *in vivo* was more likely. Our current study utilizes a 40-fold higher intravenous dose of paclitaxel (20 mg/kg). At this dose, it is conceivable that non-sink conditions likely occurred *in vivo* since plasma concentrations that were achieved using the commercial formulation (see Figure 3) clearly exceed the plasma solubility of paclitaxel (i.e., 40 μg/mL). The occurrence of non-sink dissolution conditions following intravenous administration would result in a slower dissolution rate that would not be considered ‘instantaneous.’ Our data are consistent with slowly dissolving nanoparticles being taken up into organs by phagocytic cells of the mononuclear phagocyte system that are abundant in tissues such as the liver and spleen [38,39]. One possible way to overcome the above issue is to use infusion instead of bolus injection (upon fully determining the PK/PD) to allow better dissolution of the nanoparticles, where recently, a successful use of nanoparticles to deliver drugs to high plasma concentration was reported [32]. An additional factor that may contribute to the observed difference in pharmacokinetics is that there are known non-linearities in pharmacokinetics caused by Cremophor EL impacting both paclitaxel distribution and elimination [40]. Since our nanosuspension formulation contains only a very small percentage (0.1%) of Cremophor EL compared to the standard commercial formulation, less non-linearity is expected with nanosuspension delivery.

Recently, a paclitaxel nanosuspension formulation was evaluated in a manuscript describing a pharmacokinetic study in rats and a tissue distribution study in mice [41]. Similar alterations in paclitaxel plasma clearance was observed following intravenous administration to rats but were of a lesser magnitude. In the rat study, plasma clearance was approximately 4-fold higher with nanosuspension delivery versus the 30-fold difference that we observed in our study. In the same manuscript, an evaluation of formulation-dependent changes in tissue distribution in mice was also performed. Higher tissue accumulation was reported for the liver and spleen in mice. However, it is difficult to compare results directly with our current study since plasma was not collected, and therefore, tissue to plasma ratios were not reported. Finally, non-tumor-bearing animals were used in the reported study, so there were no comparisons of tumor disposition and anti-tumor activity.

To date, to our knowledge, there have been little to no comparisons of pre-clinical anti-tumor efficacy using nanosuspension formulation to deliver anti-cancer agents to subcutaneous tumor models. In particular, investigations on the use of nanosuspension formulation for paclitaxel delivery have been limited to the pharmacokinetic/tissue distribution study that was discussed above [41]. Our current study in tumor-bearing xenograft mice clearly shows that intravenous delivery of a 20 mg/kg paclitaxel dose using nanosuspension resulted in reduced efficacy compared to the standard Cremophor EL:ethanol formulation (Figure 6). Since the plasma and tumor disposition were altered with nanosuspension delivery, anti-tumor efficacy normalized with respect to plasma and tumor exposures was calculated. The calculated measure of normalized efficacy (i.e., TGI/AUC_0-8_ ratio) provides an assessment of efficacy relative to relevant *in vivo* concentrations such that the two formulations can be properly compared. The TGI/AUC_0-8_ ratios normalized relative to plasma exposure were much higher (approximately 16-fold) for nanosuspension delivery compared to the standard formulation (Figure 7). However, the TGI/AUC_0-8_ ratios normalized relative to tumor exposure were comparable. This observation suggested that the large difference in the TGI/AUC_0-8_ ratios normalized relative to plasma exposure was a result of a higher degree of accumulation in the tumor occurring with nanosuspension delivery. Once in the tumor, paclitaxel's anti-tumor effect was similar and not dependent on the formulation. Despite having a larger tumor to plasma ratio (Table 2), nanosuspension delivery resulted in less anti-tumor efficacy (Figure 6). This occurred because the absolute amount of paclitaxel getting into the tumor was much less due to much lower plasma exposures following nanosuspension delivery (Table 1). Finally, based on the mice body weights, the tolerability of the nanosuspension formulation was acceptable.

## Conclusions

The delivery of poorly soluble drugs using nanoparticles has received much interest recently for both the oral and intravenous routes of administration. However, much of the published literature evaluates the effect of nanoparticle formulations in pharmacokinetic studies. Thus, there is a need to examine the impact of nanoparticle delivery in pre-clinical efficacy models. Our current work compares both pharmacokinetics and anti-tumor efficacy for paclitaxel delivered using a standard commercial formulation and a nanosuspension. We found that nanosuspension delivery reduced paclitaxel's anti-tumor efficacy. This, to our best knowledge, was never been investigated before. The paclitaxel dose used in our study was chosen in an attempt to match clinically relevant exposures and resulted in robust efficacy in the xenograft tumor-bearing mice when delivered with the commercial formulation. Based on our findings, the reduced anti-tumor activity associated with nanosuspension delivery appeared to be a result of non-sink dissolution conditions present at the paclitaxel dose used in our study. Finally, the current case study illustrates a need for careful consideration of both compound dose and systemic solubility prior to utilizing nanosuspension as a mode of intravenous delivery.

## Competing interests

The authors declare that they have no competing interests.

## Authors’ contributions

P-CC is PI (Pharmaceutics). SG participated in the *in vivo* efficacy studies. MN developed the *in vivo* model. AQ analyzed the BA samples. YD conducted the bioanalytical method development. AA carried out the *in vivo* experiments. KRK conducted the data collection and review. YR prepared the formulations. HW is PI (DMPK). All authors read and approved the final manuscript.
